# Sphingolipid diversity in *Candida auris*: unraveling interclade and drug resistance fingerprints

**DOI:** 10.1093/femsyr/foae008

**Published:** 2024-03-05

**Authors:** Basharat Ali, Mohit Kumar, Praveen Kumar, Anshu Chauhan, Sana Akhtar Usmani, Shivaprakash M Rudramurthy, Jacques F Meis, Arunaloke Chakrabarti, Ashutosh Singh, Naseem A Gaur, Alok K Mondal, Rajendra Prasad

**Affiliations:** Amity Institute of Integrative Science and Health and Amity Institute of Biotechnology, Amity University Gurgaon, Haryana, 122413, India; School of Life Sciences, Jawaharlal Nehru University, New Delhi, 110067, India; Amity Institute of Integrative Science and Health and Amity Institute of Biotechnology, Amity University Gurgaon, Haryana, 122413, India; Yeast Biofuel Group, International Centre for Genetic Engineering and Biotechnology, New Delhi, 110067 India; Amity Institute of Integrative Science and Health and Amity Institute of Biotechnology, Amity University Gurgaon, Haryana, 122413, India; Amity Institute of Integrative Science and Health and Amity Institute of Biotechnology, Amity University Gurgaon, Haryana, 122413, India; Department of Biochemistry, University of Lucknow, Lucknow, 226007 India; Postgraduate Institute of Medical Education and Research, Chandigarh,160012 India; Institute of Translational Research, Cologne Excellence Cluster on Cellular Stress Responses in Aging-Associated Diseases and Excellence Center for Medical Mycology (ECMM), University of Cologne, Cologne, 50931 Germany; Postgraduate Institute of Medical Education and Research, Chandigarh,160012 India; Department of Biochemistry, University of Lucknow, Lucknow, 226007 India; Yeast Biofuel Group, International Centre for Genetic Engineering and Biotechnology, New Delhi, 110067 India; School of Life Sciences, Jawaharlal Nehru University, New Delhi, 110067, India; Amity Institute of Integrative Science and Health and Amity Institute of Biotechnology, Amity University Gurgaon, Haryana, 122413, India

**Keywords:** *Candida auris*, clades, drug resistant, electrospray ionization tandem mass spectrometry, fluconazole, sphingolipids

## Abstract

In this study, we explored the sphingolipid (SL) landscape in *Candida auris*, which plays pivotal roles in fungal biology and drug susceptibility. The composition of SLs exhibited substantial variations at both the SL class and molecular species levels among clade isolates. Utilizing principal component analysis, we successfully differentiated the five clades based on their SL class composition. While phytoceramide (PCer) was uniformly the most abundant SL class in all the isolates, other classes showed significant variations. These variations were not limited to SL class level only as the proportion of different molecular species containing variable number of carbons in fatty acid chains also differed between the isolates. Also a comparative analysis revealed abundance of ceramides and glucosylceramides in fluconazole susceptible isolates. Furthermore, by comparing drug-resistant and susceptible isolates within clade IV, we uncovered significant intraclade differences in key SL classes such as high PCer and low long chain base (LCB) content in resistant strains, underscoring the impact of SL heterogeneity on drug resistance development in C*. auris*. These findings shed light on the multifaceted interplay between genomic diversity, SLs, and drug resistance in this emerging fungal pathogen.

## Teaser

*Candida auris* clades possess distinct clade and resistance specific sphingolipid fingerprints.

## Introduction

The simultaneous global emergence of distinct clades of *Candida auris*, along with its resistance to multiple antifungal drugs, has posed a significant challenge for clinicians worldwide (Lockhart et al. 2017). Compared to other *Candida* species, *C. auris* isolates found in hospitals often exhibit high levels of resistance to azoles and can display collateral resistance to amphotericin B (AmB) and echinocandins (Chowdhary et al. 2018). Azoles target the lanosterol 14-α-demethylase encoded by *ERG11* leading to accumulation of toxic sterols in the yeast cell. The commonly recorded resistance mechanism to fluconazole (FLC) includes transcriptional activation of its target *ERG11* or generating mutant variants of the target protein (White 1997, Morio et al. 2010). The increased efflux of FLC facilitated by overexpressed export proteins from the ABC or MFS families is another major determinant that contribute to azole resistance. Recently, segmental chromosomal duplications have also been linked to drug resistance in *C. auris* (Bhattacharya et al. 2019, Kim et al. 2019, Wasi et al. 2019, Rybak et al. 2021, Narayanan et al. 2022). The widespread azole resistance observed in *C. auris* has prompted researchers to investigate alternative mechanisms of drug resistance that may explain this behaviour. Understanding the complex interplay of canonical mechanisms and identifying new strategies to combat drug resistance in *C. auris* is vital (Chaabane et al. 2019).

Lipid molecules, particularly sphingolipids (SLs) and phosphoglycerides (PGLs) have gained attention as molecular determinants influencing the drug susceptibilities of yeast cells (Kohli et al. 2002, Mukhopadhyay et al. 2004). While PGLs in yeast cells share compositional similarities with those found in other eukaryotic cells, the SL profiles of fungi are unique. In mammals, SLs consist of glycosphingolipids and gangliosides, whereas yeast cells incorporate inositol and mannose, along with neutral SLs, to form complex acidic SLs (Smith and Lester 1974, Del Poeta et al. 2014, Renne and de Kroon 2018). The synthesis of SLs initiates in the endoplasmic reticulum through the condensation of serine (an amino acid) and palmitoyl Co-A (fatty acid derivative), which is catalysed by the enzyme serine palmitoyltransferase (SPT) (Levine et al. 2000, Funato and Riezman 2001). The product of this condensation reaction is 3-ketodihydrosphingosine, which is reduced to yield dihydrosphingosine (DHS). From DHS two branches originate in the pathway with different end products. The neutral branch terminates with the formation of glucosylceramides (GlcCer) while as the acidic branch terminates with the formation of mannosyl diinositolphosphorylceramide [M(IP)_2_C] (Shoma et al. 2023). Interestingly, the end product of SL biosynthesis differs among organisms. In *Saccharomyces cerevisiae*, the final product is M(IP)_2_C, which is generated through three irreversible steps and GlcCer is not synthesized by this yeast (Hanada 2003, Saito et al. 2006, Usmani et al. 2023). Lack of GlcCer synthesis has been also reported in *Candida glabrata, Candida guilliermondii*, and so on (Saito et al. 2006). In contrast, fungi from the evolutionary distinct mucormycotina such as *Mucor hiemalis, Rhizopus microsporus* have GlcCer as the primary complex SL and do not have inositol phosphorylceramides (IPCs; acidic SL) (Aoki et al. 2004). Common pathogenic fungi like *Cryptococcus neoformans* and *Candida albicans* have both the acidic and neutral pathways active (Oura and Kajiwara 2010, Singh et al. 2017, Garbe et al. 2022). The enzymes involved in the biosynthesis of complex SLs in fungi are absent in mammals, making them a potential target for new antifungal therapies (Nimrichter and Rodrigues 2011). SLs also play crucial roles in various cellular processes, including signalling, heat stress response, and serving as structural components (Dickson 2010).

SLs and ergosterol, another important lipid component, interact within the microdomain of membrane. While common antifungal drugs target ergosterol synthesis, the precise role of SLs in influencing drug resistance is beginning to emerge (Mazu et al. 2016, Rollin-Pinheiro et al. 2016, Pan et al. 2018). We have shown earlier that the deletion of erg or SLs biosynthetic genes results in increased susceptibility towards antifungals in *C. albicans*. The deletion of SLs or ergosterol also affect membrane localization of major multidrug exporter protein e.g Cdr1p in *C. albicans* (Pasrija et al. 2008) Furthermore, some SLs have been found to attenuate fungal pathogenesis. Inhibition or deletion of enzymes involved in the biosynthetic pathways of specific SLs, such as IPCs and GlcCer, have been shown to affect fungal virulence (Zhong et al. 2000, Levery et al. 2002, Rittershaus 2006). In fact, natural inhibitors of fungal GlcCer synthesis have been identified and possess antifungal properties (Mor et al. 2015).

Deletion of *IPT1*, a gene involved in the synthesis of M(IP)_2_C, in *C. albicans* and *C. glabrata* has been shown to significantly increase susceptibility to azole antifungal drugs (Prasad et al. 2005, Shahi et al. 2022). Similarly, the null mutants of *FEN1* and *FEN12*, which encode enzymes responsible for synthesizing very long-chain fatty acids, exhibit increased susceptibility to AmB in both *S. cerevisiae* and *C. albicans* (Sharma et al. 2014). Additionally, upregulation of SL biosynthesis genes has been observed in FLC resistant *C. albicans* isolates (Gao et al. 2018). Imbalances in the levels of SLs or ergosterol have been found to directly affect the trafficking of ABC efflux pump proteins, rendering *C. albicans* highly susceptible to antifungal drugs. These findings highlight the intricate interplay between intracellular drug accumulation, drug efflux mechanisms, and the membrane lipid environment in determining the drug susceptibility phenotype of *Candida* species (Bagnat et al. 2000, Urbanek et al. 2022). Understanding these interactions is crucial for developing strategies to overcome drug resistance and enhance the effectiveness of antifungal therapies.

Considering the intricate relationship between membrane lipids and drug resistance, and the limited information available regarding these aspects in *C. auris*, it is crucial to investigate the landscape of SLs and their role in supporting drug resistance. Large-scale mass spectrometry (MS)-based lipidomic studies have proven valuable in establishing connections between specific lipid structures, their levels, and physiological functions in yeast. Singh et al. (2012) showed that mitochondrial lipids are associated with cell wall integrity and azole resistance in *C. albicans* (Singh et al. 2012). By employing the ESI-MS/MS (electrospray ionization tandem MS) approach, recently, Shahi et al. (2020) presented a comparative lipidome between drug-resistant and susceptible *C. auris* isolates and pointed towards a significant remodelling of polar lipids in drug-resistant *C. auris*. In another study, Kumar et al. (2021) analysed the molecular SL signatures of drug-resistant clinical isolates of *C. auris* recovered from Indian hospitals. The study highlighted the distinct specific molecular species fingerprints of SL classes among the tested isolates, reinforcing their influence on drug resistance (Kumar et al. 2021). Together, these studies point towards the compositional remodelling of SL species structures that could be responsible for drug resistance. However, detailed studies, including a larger pool of isolates of known clades of *C. auris* recovered from different geographical regions, are required to understand the intricacies of such changes.

The present study explores the sphingolipidomic fingerprint of *C. auris* clade isolates recovered from different geographical locations. These *C. auris* strains belonging to the South Asian, East Asian, African, South American, and Iranian clades I, II, III, IV, and V, respectively, were included in the present study. The present study could not only highlight the distinct interclade fingerprints of SLs but also reveals the diversity of SL species between drug susceptible and resistant isolates of *C. auris*.

## Materials and methods

### Strains, media, and reagents

*Candida auris* strains used in this study were acquired from the National Culture Collection of Pathogenic Fungi (NCCPF), Postgraduate Institute of Medical Education and Research (PGIMER), Chandigarh (NCCPF 470156, NCCPF 470157 both isolated from blood, and NCCPF 470296, isolated from pus). CBS 10913T, the first clinical isolate of *C. auris*, was acquired from the Central Bureau voor Schimmel Cultures (CBS), Fungal Biodiversity Centre of the Royal Netherlands, Academy of Arts and Sciences (KNAW), the CDC & FDA Antibiotic Resistance Isolate Bank (AR 0383–0386, all isolated from blood and AR 1097 isolated from ear discharge). Strains VPCI 479/P/13 (isolated from blood), and LMDM 1219 (Li et al. 2021) were received as a kind gift from Prof. Dominique Sanglard, University of Lausanne and University Hospital Centre, Lausanne, Switzerland. All strains were archived in 25% glycerol at −80°C and revived on YPD agar at 30°C for experimental purposes.

All solvents and reagents used (unless specified) were LCMS grade, purchased from Honeywell NC, USA, and Sigma Aldrich MO, USA. Lipid standards were purchased from Avanti Polar Lipids Inc. AL, USA.

### Drug-susceptibility assays

Minimum inhibitory concentrations (MICs) for FLC and AmB against *C. auris* strains were determined as described by the Clinical and Laboratory Standards Institute (CLSI) by broth microdilution method with 2-fold serial dilutions in 96-well microtitre plates (M27Ed4: Broth Dilution Antifungal Susceptibility, Yeasts; CLSI 2017). For FLC, 50% reduction in growth of a particular strain compared to drug-free control (YPD) was considered as the endpoint. Whereas for AmB, 100% growth inhibition was considered as the endpoint.

### Lipid extraction

Cultures were grown in liquid YPD at 30°C till saturation. From this, a secondary culture with a starting OD_600_ of 0.1 in fresh media (50 ml) was grown up to OD_600_ 0.8–1 (mid-log phase). Approximately 5 × 10^8^ cells in three biological replicates of each strain were harvested by centrifugation at 4000 × *g* for 5 minutes. Pellets were washed twice with sterile water. Before lysis, C17 Sphingosine and C17 Ceramide (d18:1/17:0), as internal standards, were added to each pellet and then lysed using glass beads (50 mg, 0.4–0.6 mm) in Fastprep® (MP Biomedicals, CA, USA). Lipid extraction and base hydrolysis was performed using the methods described earlier by Kumar et al. (2021). Extracted lipids were dried with N_2_ flushing and stored at −20°C until analysed.

### Protein estimation

Protein estimation for normalizing lipid data was done using bicinchoninic acid (BCA) Protein Assay kit (G-Biosciences, MO, USA). From cell lysate of each replicate, an aliquot of 25 µl was added to the working solution (200 µl) in 96-well plates, and absorbance was read at 590 nm. Serial dilutions of bovine serum albumin (G-Biosciences, MO, USA) were used for standard calibration curve. The amount of protein (mg ml^−1^) was calculated from the slope of the standard calibration curve.

### Liquid chromatography MS

Extracted lipids were resuspended in organic buffer (methanol containing 1 mM ammonium formate and 0.2% formic acid). A two-buffer mobile system, aqueous and organic was used. Aqueous buffer contained 2 mM ammonium formate and 0.2% formic acid. From each sample, 5 µl was injected by the Autosampler, and mobile buffer was pumped at a flow rate of 0.3 ml min^−1^ to the HPLC fitted with the C8 column (Waters, MA, USA). SL species were detected by multiple reaction monitoring (MRM) methods using QTRAP® 4500 (SCIEX, USA) mass spectrometer. The MRM scans used were described earlier by Kumar et al. (2021).

### Data analysis and statistical analysis

Mass spectrometric chromatograms were processed using MultiQuant^TM^ software (SCIEX). Quantification of different lipid classes and species was done using the internal standard normalization method. The data was further normalized to per mg protein, and the amount of each lipid species was calculated as % of the total SL per mg protein. Three biological replicates of each sample were used for all analyses. To determine statistical significance between the data sets, Student's *t*-test was used and a *P*-value of < .05 was considered significant. PCA was performed and plotted using OriginPro^®^ software. Data bars were plotted using GraphPad Prism 8.

## Results

### All the clade isolates show susceptibility to SL biosynthesis inhibitors

Our collection of clinical *C. auris* isolates from various clades comprises three isolates from Clade I (NCCPF 470156, NCCPF 470157, and VPCI 479/P/13), two isolates from Clade II (NCCPF 470296 and CBS 10913T), two isolates from Clade III (AR 0383 and AR 0384), three isolates from Clade IV (AR 0385, AR 0386, and LMDM 1219), and one isolate from Clade V (AR 1097). Before conducting lipidomic analysis, we evaluated the susceptibility of all these isolates to SL biosynthesis inhibitors, specifically myriocin (which targets SPT) and aureobasidin A (which targets IPC synthase). Remarkably, all 11 isolates from different clades exhibited susceptibility to myriocin and aureobasidin A, with low MIC values ranging from 0.01 to 0.25 µg ml^−1^ (Table 1). This stands in contrast to *C. glabrata* and *C. albicans* reference strains, which demonstrated higher MIC values with MIC of ∼4 µg ml^−1^ against myriocin and ∼0.5 µg ml^−1^ against Aureobasidin A. (Rollin-Pinheiro et al. 2021, Kumar et al. 2021). Consequently, it can be inferred that there is no interclade heterogeneity in the susceptibility of *C. auris* isolates to SL biosynthesis inhibitors.

**Table 1.** List of clinical isolates of *C. auris* and their drug susceptibility profiles.

Clade	Isolate name	MIC (µg ml^−1^)			
		Fluconazole	Amphotericin B	Myriocin	Aureobasidin A
I	NCCPF 470156	512	1	0.06	0.015
	NCCPF 470157	512	1	0.125	0.03
	VPCI 479/P/13	256	1	0.01	0.03
II	NCCPF 470296	16	0.5	0.06	0.25
	CBS10913T	8	0.5	0.03	0.03
III	AR 0383	256	0.5	0.01	0.125
	AR 0384	128	0.5	0.01	0.01
IV	AR 0385	> 512	1	0.01	0.0625
	AR 0386	> 512	1	0.03	0.03
	LMDM 1219	16	4	0.01	0.03
V	AR 1097	128	0.5	0.03	0.03

Notably, the collection of 11 *C. auris* strains encompassed a variety of susceptible and resistance patterns to FLC and AmB. For instance, within clade I, all three isolates, namely NCCPF470156, NCCPF470157, and VPCI 479/P/13, were resistant to FLC (Table 1), while both isolates within clade II, NCCPF 470296, and CBS10913T were susceptible to FLC. Within clade III, two isolates (AR 0383 and AR 0384) demonstrated resistance to FLC. In clade IV, two out of three isolates (AR 0385 and AR 0386) displayed high MIC values against FLC (> 512 µg ml^−1^). LMDM 1219 in clade IV was susceptible to FLC but resistant to AmB (MIC 4 µg ml^−1^). All other isolates were susceptible to AmB with MIC's ranging from 0.5 to 1 µg ml^−1^. The sole isolate of clade V was FLC resistant. Importantly, regardless of their resistance profiles, all drug-resistant isolates remained susceptible to SLs inhibitors thus underscoring the importance of SL metabolism in drug resistance. It has been already shown that by targeting the SL biosynthesis pathway using inhibitors, the MIC values of FLC decreased by manifold (Rollin-Pinheiro et al. 2021). There are other studies where it has been demonstrated that creating blocks at specific steps in the SL biosynthesis alters the susceptibility of fungal cells against the common antifungal drugs such as FLC (Oura and Kajiwara 2010).

### The five clades of *C. auris* exhibit unique landscapes of SLs, and notably, both the neutral and acidic SL biosynthesis pathways are active in all of them

**Figure 1.**
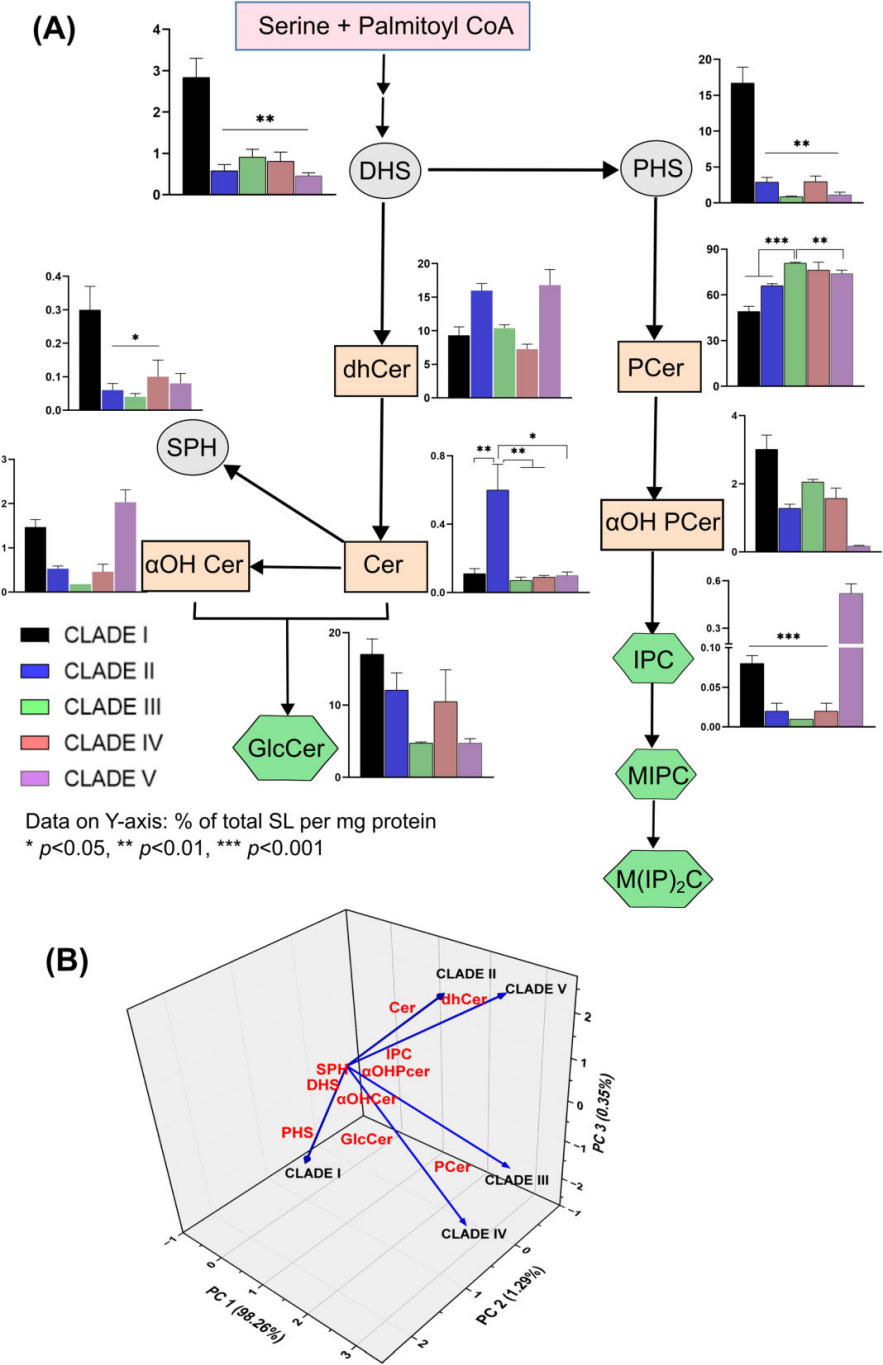
SL class composition differentiates the geographical clades. (A). SL biosynthesis pathway of *C. auris* and levels of different SL intermediates (mean + SEM) in each of the five clades as detected by ESI-LCMS/MS. Data on *Y*-axis represents % of total SL per mg protein). Clades showing significant variations are marked * *P* < .05, ** *P* < .01, and *** *P* < .00. DHS—dihydrosphingosine, PHS—phytosphingosine, dhCer—dihydroceramide, Cer—ceramide, αOH Cer—alpha hydroxyl ceramides, αOH PCer—alpha hydroxyl phytoceramides, GlcCer—glucosylceramides, and IPC—inositol phosphorylceramides. Ovals represent the three major sphingoid bases (18-carbon long amino-alcohol), the rectangles represent the SL classes derived from fatty acylation of sphingoid bases. Hexagons represent the complex SLs having a head group at C1 position of the sphingoid base. (B) 3D PCA biplot showing separation of five clades of *C. auris* based on the composition of different SL classes. Vectors represent the projection of clades based on loading values on three axes PC1, PC2, and PC3. SL classes (red) occupy positions in the plot based on scores achieved in PCA.

We were able to identify and quantify various classes of SLs and their respective species in *C. auris* isolates from different clades using MRM based LC-MS analysis. The *C. auris* SLs we identified fall into eight main classes: sphingoid bases (LCBs), dihydroceramides (dhCer), ceramides (Cer), α-hydroxyceramides (αOH Cer), phytoceramides (PCer), α-hydroxyphytoceramides (αOH PCer), GlcCer, and IPC (Fig. 1A). Additionally, the sphingoid bases were further categorized into seven distinct subclasses: DHS, sphingosine (SPH), sphingosine-1-phosphate (S1P), dihydrosphingosine-1-phosphate (DHS1P), phytosphingosine (PHS), phytosphingosine-1-phosphate (PHS1P), and glucosyl sphingosine (Glucosyl-SPH). It is worth noting that we could not quantify phosphorylated long chain bases (LCBs), mannosylated SLs (MIPC and M(IP)_2_C), and glucosyl-SPH, likely due to their lower abundance.

Our analysis confirmed that both the acidic and neutral branches of the SL biosynthesis pathway are active across all *C. auris* clades. This observation aligns with *C. albicans* and separates *C. auris* from other fungi as discussed above.

Our analysis revealed distinct characteristics in different clades. Clade I stood out due to its notably higher levels of three sphingoid bases (DHS 2.84%, PHS 16.71%, and SPH 0.3%) and GlcCer (17.02%) when compared to the other three clades (Fig. 1A). Conversely, clade II exhibited the highest Cer content at 0.6%. Clade III, on the other hand, displayed the highest PCer content (∼80%) but had lower levels of PHS and αOH Cer (0.8% and 0.18%, respectively) compared to the other clades. In contrast, Clade V showed the highest dhCer content at 16.79%, while it was the least abundant in Clade IV isolates at 7.25% compared to all the clades. Notably, Clade V also had higher levels of αOH Cer (2.03%) and IPCs (0.52%). It's worth mentioning that IPC levels, representing the acidic SL biosynthesis pathway, were only detected in trace amounts, except for Clade V, which exhibited characteristically high levels of IPC (Fig. 1A).

To assess whether these variations in SL class composition effectively distinguish the five clades, we conducted principal component analysis (PCA) on the respective SL datasets. As depicted in Fig. 1(B) (File S1, Supporting Information), the PCA plot distinctly separated each clade from the others. PCA analysis extracted three principal components, namely PC1, PC2, and PC3, which together accounted for 99.9% of the variance within the data. Clade assignments were based on PCA loading values, and SL class assignments were based on scores (Fig. 1B). Clades I, II, and V displayed noticeable differences when considering the scores depicted in the plot. In contrast, Clades III and IV appeared to be more closely positioned on the plot due to their relatively similar SL profiles. A similar close positioning was also observed in Clade II and V in the plot due to similar content of some SL classes. Specifically, PCer and PHS exhibited the highest scores on PC1 and PC2, aligning with the loading values associated with Clades III and I, respectively. On the other hand, dhCer attained a high score on PC3 and corresponded with Clades II and V, effectively distinguishing these two clades from others (Fig. 1B).

### The distribution of SL classes varies among the *C. auris* isolates

**Figure 2.**
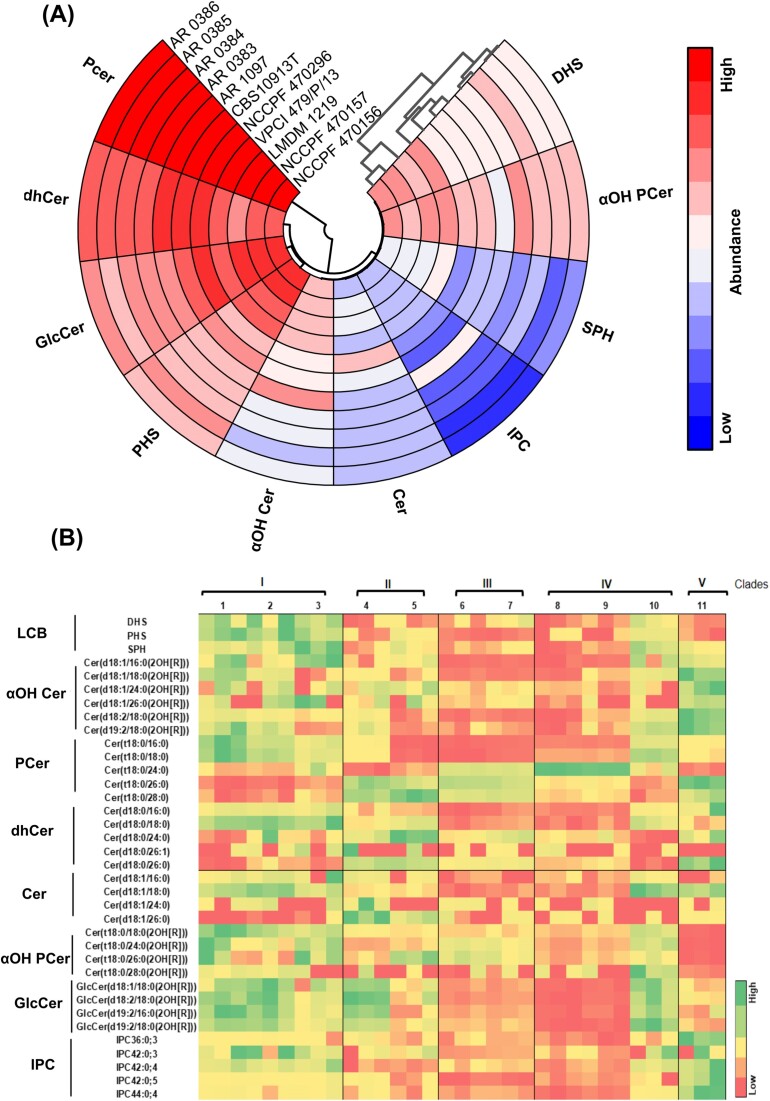
SL distribution in *C. auris* isolates. (A) Polar heatmap showing SL class composition in the eleven clinical isolates of *C. auris*. The colour gradient is based on log_10_ scale of the lipid proportion of each class in all isolates. (B) Heat map depicting variation of SL molecular species, which were abundant and differed highly between the isolates numbered on top 1–11: NCCPF470156, NCCPF 470157, VPCI 479/P/13, NCCPF 470296, CBS10913T, AR 0383, AR 0384, AR 0385, AR0386, LMDM 1219, and AR 1097, respectively. Three replicates of each isolate are shown in the heatmap.

To delve deeper into the differences in the composition of SL classes among the isolates, we performed a comprehensive analysis that included all 11 isolates representing various clades, regardless of their drug-susceptibility profiles. Out of the seven SL classes and three sphingoid bases, PCer emerged as the predominant class in all 11 isolates. Notably, isolates from clade IV, specifically AR 0385 and AR 0386, exhibited the highest PCer content among all the isolates, with percentages of 86.4% and 86.1%, respectively (Fig. 2A; File S2, Supporting Information). The second most abundant SL class across six isolates from clades II, III, IV, and V was dhCer, including CBS10913T (18.14%) from clade II, AR 0383 (9.3%) and AR 0384 (11.3%) from clade III, two isolates from clade IV, AR 0385 (8.4%) and AR 0386 (8.7%), and clade V isolate AR 1097 (16.7%). In contrast, GlcCer emerged as the second most abundant SL class in LMDM 1219 (27.4%), NCCPF 470156 (21.16%), NCCPF 470157 (20.17%), and NCCPF 470296 (17%). PHS was the predominant sphingoid base in all isolates across various clades. Notably, clade I isolates, including NCCPF 470156, NCCPF 470157, and VPCI 479/P/13, displayed relatively higher PHS levels, respectively accounting for 19.7, 17.8, and 12.4% of the SL content when compared to isolates from other clades. Following these, LMDM 1219 (5.77%) and CBS10913T (3.96%) also showed notable PHS levels, while AR 0383 and AR 0384 exhibited the lowest PHS levels at ~0.85%. DHS, another sphingoid base, exhibited significant variation among the isolates. It was most abundant in three clade I isolates (ranging from 2.4% to 3.4%), followed by LMDM 1219 (1.5%) and AR 0384 (1.1%). For all other isolates, DHS levels were less than 1%. αOH Cer content was highest in the clade V isolate (2%), followed by clade I isolates and LMDM 1219, with levels ranging from 1.1% to 1.9%. In contrast, all other isolates exhibited αOH Cer levels of less than 1%, with the lowest levels found in strains AR 0383, AR 0384, AR 0385, and AR 0386, at 0.09%–0.19%. αOH PCer content was least abundant in the clade V isolate (0.17%) and AR 0385 (0.8%), while all other strains had content ranging from 1% to 3%. As observed in the polar heat map (refer to Fig. 2A), SL classes such as SPH, Cer, and IPC were among the least abundant across all clades. NCCPF 470296 and CBS10913T had the highest Cer content, whereas its levels were notably low in isolates from clade III and two isolates from clade IV (AR 0385 and AR 0386). SPH was enriched in three clade I isolates and LMDM 1219, with both classes constituting less than 1% of the SL content. IPCs were detected in low amounts, except in the clade V isolate, where they were remarkably high (0.52%) compared to all other isolates.

### Molecular species of SL classes display different profiles among clade isolates

To check how this variation of SL classes is constituted in each strain, we analyzed the molecular species of SL classes that contribute to this variation. Every molecular species is a unique structure and differ from one another in the backbone, fatty acyl chain length, degree of saturation, type of head group and number of hydroxyl groups present (Usmani et al. 2023). Throughout different classes, fatty acyl chains having carbon chain length of 12–30 were detected in our analysis. However, not all contributed equally to the overall lipid content showing that only few are preferred over others for acylation. Fatty acids with carbon chain lengths of 16, 18, 24, 26, and 28 showed predominance in different classes and saturated fatty acids were highly preferred over the unsaturated ones (File S3, Supporting Information). Except GlcCer, most of the SL classes had high abundance of molecular species containing 24, 26, and 18C long fatty acids followed by ones having 28 and 16 carbons (Fig. 2B). In GlcCer, the 18C long fatty acid containing specie, GlcCer(d19:2/18:0(2OH))] is remarkably very high followed by 16C containing GlcCer(d19:2/16:0(2OH))]. Also, this class contained species with different backbones like d18:1, d18:2, and d19:2 coming from different pools of Cer and αOH Cer. GlcCer(d19:2/18:0(2OH[R])) turned out to be the major species followed by GlcCer(d18:2/18:0(2OH[R])) and GlcCer(d19:2/16:0(2OH[R])). This trend remained uniform across all the isolates. The upstream precursor classes of neutral branch such as dhCer, Cer, and αOH Cer had abundance of 16C, 18C, 24C, and 26C fatty acids. In dhCer, Cer(d18:0/24:0) was major dhCer specie across all isolates followed by Cer(d18:0/18:0) or Cer(d18:0/26:0). In Clade II and Clade V isolates, Cer(d18:0/26:0) was the second most abundant species while as in other isolates it was Cer(d18:0/18:0) (Fig. 2B). Unlike the neutral branch, very long chain fatty acids (VLCFA) having 24C and 26C are more preferred in the acidic pathway compared to 28C and 18C ones. As already mentioned above that PCer is major SL class in all isolates (Fig. 2A), but the proportion of PCer molecular species is not uniform. In eight isolates of clade I, III, and IV the ratio of 24C PCer to 26C PCer [Cer(t18:0/24:0):Cer(t18:0/26:0)] was high (1.3–4) due to high prevalence of 24C fatty acid while as the same ratio in isolates of Clade II and V was (0.6–1). A similar trend was also observed in case of αOH PCer where the ratio of Cer(t18:024:0(2OH[R])):Cer(t18:0/26:0(2OH[R])) was high (>15) in clade I, III, and IV compared to clade II and V (< 12.5) (File S3, Supporting Information).

### Drug-susceptible and drug-resistant isolates of all clades exhibit discernible differences in their molecular species profiles

As previously mentioned, our sample collection primarily consisted of a mix of drug-susceptible and drug-resistant isolates across various clades. However, an exception was observed in clade IV, where we obtained two drug-resistant and one drug-susceptible isolate. This diversity in susceptibility profiles presented a challenge when directly comparing sphingolipidomic patterns between drug-susceptible and drug-resistant isolates from different clades. Nevertheless, we proceeded with sphingolipidomic analysis on all 11 isolates encompassing various clades and conducted comparisons between three susceptible and eight resistant isolates across the clades. The primary objective was to pinpoint specific variants within SL classes and species between the available pool of resistant and susceptible isolates.

**Figure 3.**
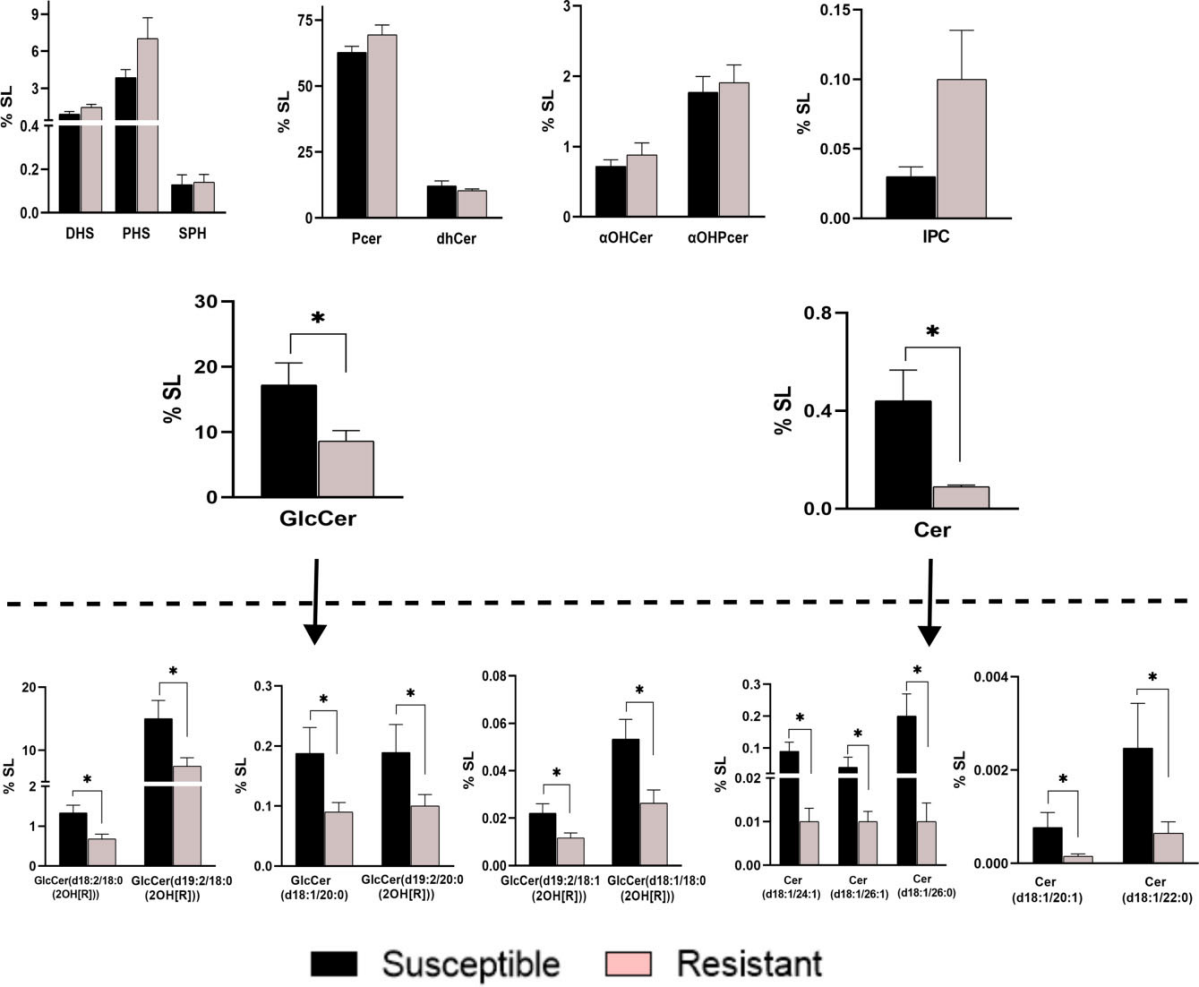
Comparative analysis of drug-resistant and -susceptible isolates. Variation in SL classes between drug-resistant and -susceptible isolates (upper panel) and molecular species that display statistically significant variation between the two groups (lower panel). Bars represent mean (+SEM) value of given lipid classes or molecular species in drug-susceptible or drug-resistant isolates. Data on *Y*-axis represents % of total SL per mg protein (*P < .05).

Upon comparing average SL class contents between the two groups, many classes showed noticeable differences between them (Fig. 3). However, GlcCer and Cer displayed statistically significant differences between the two groups. On average, both GlcCer and Cer levels were higher in the drug susceptible group. A close inspection revealed six such species form GlcCer and five from Cer contributed to this significant variation. The levels of all these species were distinctly higher in drug susceptible isolates. The major GlcCer specie GlcCer(d19:2/18:0(2OH[R]) along with GlcCer(d18:1/20:0) and GlcCer(d18:1/18:0(2OH[R])) were ∼2-fold higher and other GlcCer species were ∼1.9 fold higher in the susceptible group. Cer, although present in minute quantities showed comparatively more variation with Cer(d18:1/26:0) being more than 16-fold higher in the susceptible strains compared to the resistant ones. Other Cer species were 3–6-fold higher. The other SL classes that did not show significant differences between the two groups, but still had some molecular species that showed significant variation between the two groups.

### Different SL profiles are evident in both resistant and susceptible isolates within Clade IV

**Figure 4.**
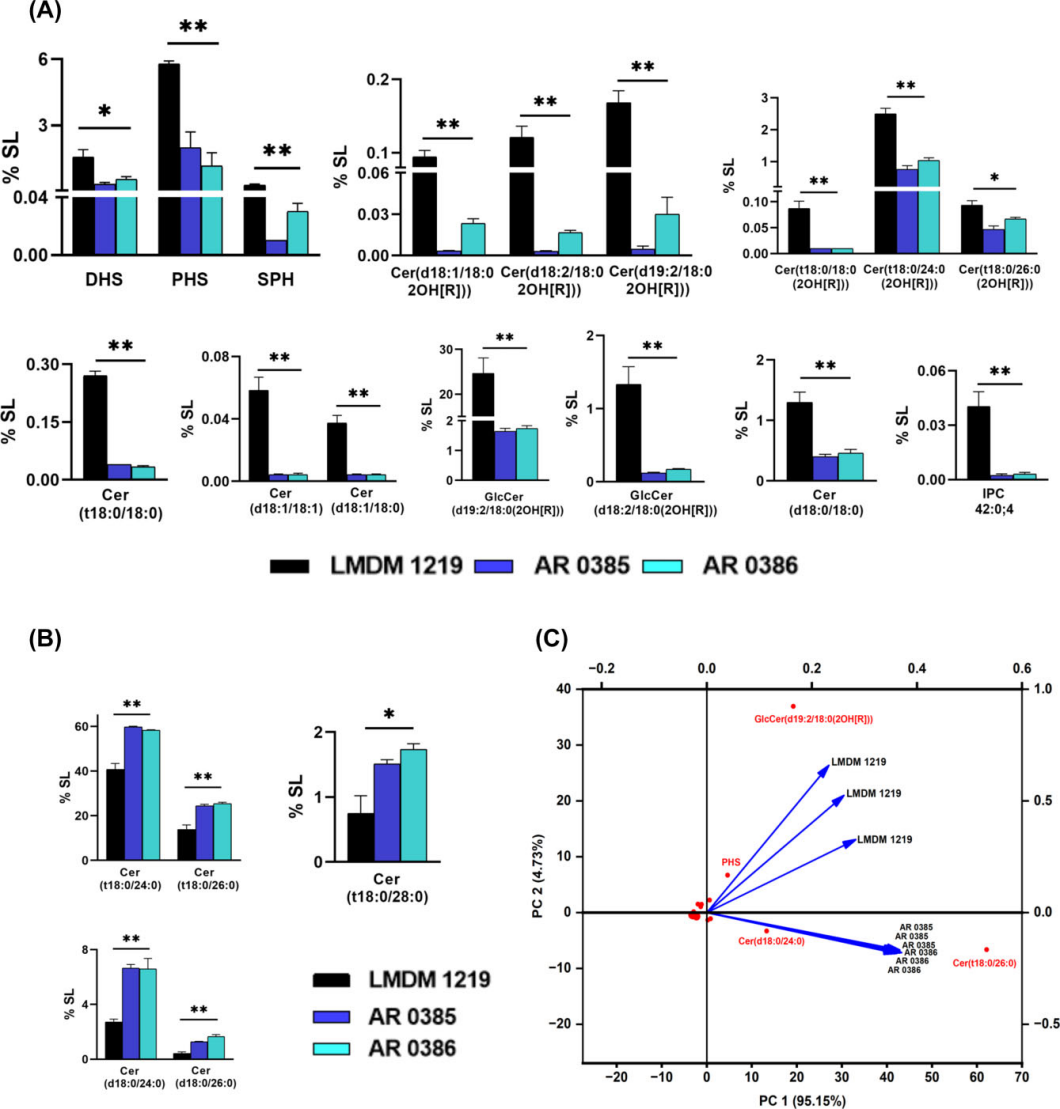
Clade IV drug-susceptible and -resistant strains are differentiated by specific lipid imprints. Bar graphs representing the molecular species that were abundant and showed statistically significant differences (*P < .05, **P < .01) between the drug-susceptible and -resistant isolates of clade IV. Each bar represents the mean + SEM of three biological replicates. Data on the *Y*-axis represents % of total SL per mg protein) (A) Molecular species within different classes that were abundant in the drug susceptible isolate LMDM 1219. (B) Molecular species that were abundant in drug-resistant isolates AR 0385 and AR 0386. (C) PCA biplot showing separation of the drug-susceptible isolate from the resistant isolates based on the overall lipid composition of three isolates of clade IV. Vectors (arrows) represent projection of isolates based on loading values obtained on PC1 and PC2. Red dots show position of molecular species in the plot based on PCA scores.

Clade IV comprises two strains resistant to FLC (AR 0385 and AR 0386) and one isolate susceptible to FLC (LMDM 1219) (Table S1, Supporting Information). This combination provided an opportunity to compare the SL profiles within Clade IV. We conducted a comprehensive analysis to identify statistically significant SL variations between the susceptible and resistant isolates within this clade. The FLC susceptible isolate had higher Cer and GlcCer levels than the two resistant isolates, consistent with our observations when comparing all 11 susceptible and resistant isolates. This similarity extended to the molecular species level as well. In the case of GlcCer, all the detected species exhibited significant variations between the two groups, with each species showing an abundance in the susceptible strain. However, most of the GlcCer content was contributed by only two species with d18:2 and d19:2 backbone acylated to α-hydroxy 18:0 fatty acid, i.e. GlcCer(d18:2/18:0(2OH[R])) and GlcCer (d19:2/18:0(2OH[R])), which were 1.3% and 24.6%, respectively, in the susceptible strain compared to 0.12%–0.17% and 1.6% –1.7%, respectively, in resistant isolates. Numerous species of GlcCer, such as (d18:1/18:0(2OH[R])), (d18:2/18:0(2OH[R])), (d19:2/18:1(2OH[R])), (d19:2/18:0(2OH[R])), and (d19:2/20:0(2OH[R])), were consistently abundant in susceptible isolates, both across different clades and within Clade IV. Notably, all three sphingoid bases, PHS, DHS, and SPH, were less abundant in the two FLC resistant strains compared to the susceptible strain (Fig. 4A). In the susceptible strain, PHS, DHS, and SPH constituted 5.7%, 1.5%, and 0.2%, respectively, while in the resistant strains, these levels ranged from 1.1%–1.9%, 0.3%–0.5%, and 0.01%–0.03%, respectively.

As previously mentioned (File S3, Supporting Information), the majority of SLs consist of molecules with 18, 24, 26, and 28 carbon-long fatty acids. A similar pattern was seen in Clade IV group of isolates. However, molecular species with 18:0 fatty acids were more abundant in FLC susceptible strains compared to their resistant counter parts. For example, in PCer and dhCer, Cer(t18:0/18:0) and Cer(d18:0/18:0) were prevalent in the susceptible strain (0.27% and 1.29%, respectively) as opposed to the resistant strains (0.04% and 0.4%–0.46%) (Fig. 4A). Very long-chain fatty acids with 24C and 26C from the same two classes and 28C PCer were relatively more abundant in resistant strains (Fig. 4B). Cer(t18:0/24:0), Cer(t18:0/26:0), and Cer(t18:0/28:0) with levels ranging from 58% to 59%, 24% to 25%, and 1.5% to 1.7% in resistant strains were comparatively higher than in the susceptible counterpart (40.7%, 13.9%, and 0.74%, respectively).On average, the level of many such species was also higher in all resistant isolates including clade IV such as Cer(t18:0/24:0), Cer(t18:0/24:1), as compared to the susceptible isolates In the case of dhCer, molecular species like Cer(d18:0/24:0) and Cer(d18:0/26:0) were relatively scarce in the susceptible strain (2.7% and 0.4% respectively) compared to the two resistant strains (6.6% and 1.2%–1.66%). In the case of Cer, two species, Cer(d18:1/18:0) and Cer(d18:1/18:1), and in αOH Cer, Cer(d19:2/18:0(2OH[R])) structures were more abundant in the susceptible strain (0.04%, 0.06%, and 0.09%, respectively) as compared to resistant strains. All other major species belonging to αOH PCer were abundant in the susceptible strain (Fig. 4A).

To emphasize the statistical significance of these variations, we conducted PCA on the respective datasets, allowing for the extraction of three components. When plotting the two primary components, PC1 and PC2, which accounted for 99.8% of the variance in the data, a clear differentiation in SL fingerprints between the susceptible and resistant strains emerged (Fig. 4C). The scores demonstrated that molecular species such as GlcCer(d19:2/18:0(2OH[R])) and PHS were abundant and aligned with the loading values of the susceptible strain, effectively separating it from the resistant strains, which were characterized by higher levels of VLCFA containing dhCer, PCer, and others (Fig. 4C; File S4, Supporting Information). Notably, among the abundant species, 21 SL species displayed statistically significant differences between the susceptible isolate and all two resistant isolates of clade IV (Fig. 4).

## Discussion

The simultaneous appearance of *C. auris* in multiple geographical areas as different clades and the observed phenotypic and genetic differences between them with respect to pathogenicity and resistance to different antifungal drugs demands a closer look (Muñoz et al. 2018, Welsh et al. 2019). Why *C. auris* manifests an exceptionally high frequency and level of resistance towards common antifungals and why some of the clades do not show a high percentage of drug resistance are some open questions that remain unanswered. *C. auris* has evolved in different geographical niches (Chow et al. 2020), which can result in dissimilarity in the membrane chemical architecture, and since membrane lipids are also the target of common antifungals wherein, slight imbalances in lipid homeostasis impact the development of resistance. The different clades of *C. auris* are believed to have independent evolutionary history. Clades have unique genomic, transcriptomic, and metabolomic signatures that differentiate the clades.(Chow et al. 2020, Brandt et al. 2023). Genomic analysis of the isolates belonging to different clades reveals Clade II to be the evolutionary oldest while as clade IV to be the recent one (Chow et al. 2020). We hypothesize that the different drug-susceptibility profiles may be attributed to dissimilar membrane lipid profiles particularly the SLs, as these play multiple roles in yeast cells including stress tolerance and drug resistance.

The present work was planned to highlight the total landscape of SLs among different clades and drug susceptible and resistant isolates. For this, by employing the ESI-based LC-MS/MS technique, we compared the sphingolipidomes of five geographical clades consisting of eleven clinical isolates of *C. auris*. Our analysis could identify lipid structures belonging to all major lipid classes of SL and show that all five clades possess composition distinct enough to separate them by multivariate analysis. In addition, these variations were reflected at the level of molecular species, which differed in fatty acid chain lengths and the sphingoid backbone as well. Among different SLs, PCer levels emerged as the most varying SL class among all the clades, while SPH was the least varying class of SL. Clade I showed maximum variation compared to other clades and the highest dissimilarity against clade II isolates. All other clades also exhibited differences but were restricted to fewer SL classes. However, in addition to the prominent characteristics, there was also a basic level of uniformity for the distribution of the various classes. For instance, PCer was uniformly abundant SL class in all 11 isolates, followed by dhCer or GlcCer, implying the importance of the large SL molecules in the architecture of the membranes in all the clades. Similar results with a higher amount of PCer were also found in our previous study with *C. auris*, and with *C. neoformans* and *C. gattii* strains. PCer has been shown to be critical in maintaining an appropriate plasma membrane environment for the proper functioning of membrane proteins in *C. neoformans* (Farnoud et al. 2014, Kumar et al. 2021). Considering their abundance levels, it is likely that PCer is also structurally indispensable in *C. auris*.

A comparison of worldwide isolates has also brought attention to clade-specific variance in drug resistance levels and resistance-related mechanisms. While most isolates from clade II are usually susceptible to azoles and other antifungals, almost all isolates from clades I, III, and more than half of the isolates from clade IV are resistant to azoles (Lockhart et al. 2017, Chowdhary et al. 2018, Chow et al. 2020). We aimed to exploit the diverse resistance profile of *C. auris* to search for lipid fingerprints associated with resistance that could be unique to the different clades. However, the unavailability of drug-susceptible isolates in all the clades and resistant isolates in Clade II was a major drawback. Nevertheless, a broad comparison of the susceptible and resistant isolates across the clades provided some insights. For example, there was higher abundance of total Cer and GlcCer in the FLC susceptible isolates compared to the resistant ones. The same distinction was also observed, with respect to GlcCer, in clade IV, where the intraclade comparison of FLC susceptible and resistant isolates was possible, suggesting the yet unexplored roles of these lipid molecules in determining the susceptibility. Variations in common species within GlcCer between two sets, may likely establish GlcCer as another marker of resistance development in *C. auris*. Apart from these common fingerprints, many other molecules showed variation in Clade IV intraclade comparison and in cross clade comparison. Perturbation of these unique lipid species could be associated with FLC resistance, provided such profiling is done on a large scale. The variation based on carbon chain length in fatty acids in respective SL classes also hints at the roles of fatty acyl elongases. Previous studies have shown that the deletion of *FEN1*/*SUR4* can lead to AMB susceptibility. *FEN1/12* are used to synthesize VLCFA in yeasts (Sharma et al. 2014). In clade IV, the FLC-resistant isolates also showed an accumulation of VLCFA containing SLs and could indicate SLs’ role in the resistant trait of *C. auris*. In addition, FLC resistant strains of clade IV had reduced levels of some GlcCer species, which were also observed previously in FLC resistant strains of clade I (Kumar et al. 2021). Altogether, clades can be separated based on the high and low abundance of different classes of SLs. Various transcriptomic and metabolomic data in clinical and FLC-adapted *C. auris* show dysregulation in SL-related genes and alteration in SL intermediates, strengthening the role of SLs in establishing *C. auris*-resistant traits (Zamith-Miranda et al. 2019, Jenull et al. 2021, Narayanan et al. 2022).

At this stage, it is difficult to comment on the physiological relevance of compositional variations among SLs. The level of dissimilarity in the sphingolipidome between the five clades points towards another level of diversity, suggesting separate levels of SL-mediated regulation in different clades. It would be interesting to explore whether these variations control clade specific azole resistance profiles. For instance, the high level of LCBs like PHS in clade I drug-resistant isolates could be a major determinant. LCBs have been shown to get induced during stress (Vandenbosch et al. 2012). The major sphingoid base PHS is known to activate different protein kinases in the cell such as Pkh1/2 and its downstream effectors Ypk1/2, Pkc1, and Sch9 (Liu et al. 2005). Pkc1 via MAPK cascade mediates FLC tolerance in *C. albicans* (LaFayette et al. 2010). Similarly in Clade III and IV, FLC-resistant isolates recorded very high PCer levels in our analysis. PCer is synthesized by acylation of PHS by ceramide synthases. Deletion of ceramide synthase *LAG1* increased susceptibility of *C. albicans* towards FLC (Gao et al. 2018). Ceramide-activated protein phosphatases such as Sit4 have been shown for modulating multidrug resistance in yeast via upregulation of PDR genes (Miranda et al. 2010). All such multilayered regulatory networks and others mediated by SLs could possibly be responsible for different resistant profiles of *C. auris* clades. Our analysis is limited to only 11 isolates; hence more in-depth analysis of sphingolipidomes with more isolates will be required to establish apparent dissimilarities among clades. Moreover, the relevance of characteristics of SLs in different clades coming from diverse niches and among drug-susceptible and -resistant isolates will require a more extensive analysis for its relevance and validation. Nonetheless, based on drug susceptibility assays using myriocin and aureobasidin A, it can be inferred that *C. auris* cannot survive without SL synthesis. It also implies that probably no other lipid classes can compensate for the loss of SL structures in myriocin or aureobasidin A-treated cells, which makes the SL biosynthetic pathway a prime target to treat *C. auris* infections.

## Supplementary Material

foae008_Supplemental_Files
